# Large Nationwide Outbreak of Invasive Listeriosis Associated with Blood Sausage, Germany, 2018–2019 

**DOI:** 10.3201/eid2607.200225

**Published:** 2020-07

**Authors:** Sven Halbedel, Hendrik Wilking, Alexandra Holzer, Sylvia Kleta, Martin A. Fischer, Stefanie Lüth, Ariane Pietzka, Steliana Huhulescu, Raskit Lachmann, Amrei Krings, Werner Ruppitsch, Alexandre Leclercq, Rolf Kamphausen, Maylin Meincke, Christiane Wagner-Wiening, Matthias Contzen, Iris Barbara Kraemer, Sascha Al Dahouk, Franz Allerberger, Klaus Stark, Antje Flieger

**Affiliations:** Robert Koch Institute, Wernigerode, Germany (S. Halbedel, M.A. Fischer, A. Flieger);; Robert Koch Institute, Berlin, Germany (H. Wilking, A. Holzer, R. Lachmann, A. Krings, M. Meincke, K. Stark);; German Federal Institute for Risk Assessment, Berlin (S. Kleta, S. Lüth, S.A. Dahouk);; Freie Universität Berlin, Berlin (S. Lüth);; Austrian Agency for Health and Food Safety, Vienna, Austria (A. Pietzka, S. Huhulescu, W. Ruppitsch, F. Allerberger);; European Centre for Disease Prevention and Control, Stockholm, Sweden (A. Krings, M. Meincke);; Institut Pasteur, Paris, France (A. Leclercq);; Ministry for Environment, Agriculture, Conservation and Consumer Protection of the State of North Rhine-Westphalia, Düsseldorf, Germany (R. Kamphausen);; State Health Office Baden-Wuerttemberg, Stuttgart, Germany (M. Meincke, C. Wagner-Wiening);; Chemical and Veterinary Investigations Office, Fellbach, Germany (M. Contzen);; Bavarian Health and Food Safety Authority, Oberschleißheim, Germany (I.B. Kraemer);; Rheinisch-Westfälische Technische Hochschule, Aachen, Germany (S.A. Dahouk)

**Keywords:** Listeriosis, foodborne diseases, outbreak, public health, molecular surveillance, whole-genome sequencing, Germany, food safety

## Abstract

Invasive listeriosis is a severe foodborne infection in humans and is difficult to control. Listeriosis incidence is increasing worldwide, but some countries have implemented molecular surveillance programs to improve recognition and management of listeriosis outbreaks. In Germany, routine whole-genome sequencing, core genome multilocus sequence typing, and single nucleotide polymorphism calling are used for subtyping of *Listeria monocytogenes* isolates from listeriosis cases and suspected foods. During 2018–2019, an unusually large cluster of *L. monocytogenes* isolates was identified, including 134 highly clonal, benzalkonium-resistant sequence type 6 isolates collected from 112 notified listeriosis cases. The outbreak was one of the largest reported in Europe during the past 25 years. Epidemiologic investigations identified blood sausage contaminated with *L. monocytogenes* highly related to clinical isolates; withdrawal of the product from the market ended the outbreak. We describe how epidemiologic investigations and complementary molecular typing of food isolates helped identify the outbreak vehicle.

Listeriosis is a severe, mainly foodborne, human infection associated with higher case-fatality and hospitalization rates than other bacterial gastrointestinal pathogens (*1*). The causative agent, *Listeria monocytogenes*, occurs ubiquitously in the environment and disseminates into the food production chain. Patients develop either self-limiting noninvasive gastroenteritis or invasive listeriosis (*2*,*3*). Listeriosis adversely affects older and immunocompromised persons, as well as pregnant women, causing a severe invasive form of the disease that leads to sepsis, meningitis, and encephalitis, as well as neonatal infections and miscarriage (*4*). Case-fatality rates of invasive listeriosis are ≈30% for neurolisteriosis and even higher in septic patients (*5*). In Europe and North America, invasive listeriosis affects 0.3–0.6 persons/100,000 population/year (*6*,*7*).

*L. monocytogenes* forms hard-to-remove biofilms in food-processing plants, can acquire tolerance to sanitizers, and multiplies even at temperatures used for refrigeration (*8*). These properties complicate efficient prevention of *L. monocytogenes* contaminations in different types of ready-to-eat products, including dairy, meat, and fish, and in fruits and vegetables, all of which have been vehicles for listeriosis outbreaks in the past (*9*–*12*).

Outbreaks of listeriosis are difficult to control for several reasons. First, case numbers are low, impairing the generation of valid hypotheses about possible food sources through patient interviews. Second, incubation time can be long, 1–67 days (*13*), and patients often are seriously ill, further complicating patient interviews. Third, the large variety of possible food sources makes pinpointing through patient interviews and follow-up tracing of food difficult. Moreover, listeriosis outbreaks can be geographically widespread due to long-distance food trade connections, e-commerce, and travel, thus hampering outbreak recognition by local authorities (*10*,*14*,*15*). In addition, listeriosis outbreaks can be protracted and last for several years (*16*), making it difficult to correctly identify affected patient groups and the common source of infection.

Nationwide systematic collection of *L. monocytogenes* isolates from human listeriosis cases and subtyping by using high-resolution whole-genome sequencing (WGS)–based typing techniques can aid in rapid and reliable detection of outbreak clusters (*3*,*17*–*22*), some of which were not detectable in the past. At the same time, systematic and on-demand typing of food-associated *L. monocytogenes* isolates assist in detecting outbreak sources. In a recent molecular surveillance study in France, one third of all isolates were grouped into WGS clusters, and most clusters contained <5 isolates (*20*). Larger outbreaks of invasive listeriosis occur, although infrequently, and 2 of the world’s largest outbreaks in the recent past included 147 cases in a multistate outbreak associated with cantaloupes in the United States in 2011 (*10*) and 1,060 cases in an outbreak associated with French polony sausage in South Africa during 2017–2018 (*11*). Since August 2019, Spain has been experiencing another large listeriosis outbreak (*23*), but the scientific evaluation of this outbreak is ongoing.

We describe an exceptionally large nationwide outbreak that included 134 laboratory-confirmed *L. monocytogenes* isolates from 112 patients with epidemiologic investigations and complementary WGS-based typing of food isolates identifying the outbreak vehicle. This outbreak represents one of the largest outbreaks of invasive listeriosis in Europe documented in the scientific literature during the past 25 years.

## Methods and Materials

### Isolation, Growth, and Serotyping of *L. monocytogenes*

We isolated *L. monocytogenes* from 184 specimens from human cases and food sources (Appendix Table 1). We performed routine *L. monocytogenes* cultures in brain heart infusion (BHI) broth, or on BHI or sheep blood agar plates at 37°C. We detected and enumerated *L. monocytogenes* from food samples according to International Organization for Standardization (ISO) methods EN ISO 11290–1:2017 and EN ISO 11290–2:2017 (*24*,*25*). We confirmed the species by using EN ISO 11290–1:2017 or matrix-assisted laser desorption/ionization time-of-flight mass spectrometry, and a previously described multiplex PCR (*26*). We used the GenElute Bacterial Genomic DNA Kit (SigmaAldrich, https://www.sigmaaldrich.com) or the QIAamp DNA Mini Kit (QIAGEN, https://www.qiagen.com) to isolate chromosomal DNA and determined molecular serogroups by using multiplex PCR (*27*).

### Genome Sequencing, Multilocus Sequence Typing, and Core Genome MLST

We quantified DNA by using the Qubit dsDNA BR or HS Assay Kit and Qubit fluorometers (Invitrogen, https://www.thermofisher.com). We prepared libraries by using the Nextera XT DNA Library Prep Kit (Illumina, https://www.illumina.com) and sequenced isolates on MiSeq, HiSeq, or NextSeq sequencers (Illumina). We trimmed and assembled raw reads in SeqSphere (Ridom, https://www.ridom.de) by using the Velvet assembler. We extracted in silico serogroups, multilocus sequence types (STs), and 1,701 locus core genome multilocus sequence typing (cgMLST) complex types (CTs) by using SeqSphere and automated allele submission to the *L. monocytogenes* cgMLST server (http://www.cgmlst.org/ncs/schema/690488) (*28*). We deposited genome sequences in the European Nucleotide Archive (https://www.ebi.ac.uk/ena; accession numbers in Appendix Table 1). Coverage ranged between 22- and 116-fold (median 54-fold). We defined cgMLST clusters as groups of isolates with <10 different alleles between neighboring isolates. We used SeqSphere in the pairwise ignore missing values mode and an unweighted pair group method with arithmetic mean to generate phylogenetic trees.

### Single-Nucleotide Polymorphism–Based Alignments

We used pipelines developed in-house to map sequencing reads, generate consensus sequences, calculate alignment, and filter single-nucleotide polymorphisms (SNPs) using an exclusion distance of 300 bps (*17*). We used the 10-092876-0769 LM12 genome (GenBank accession no. CP019625) a member of serogroup IVb, ST6, and CT6304, as the reference. We generated maximum likelihood trees by using the Geneious 9.1.3 Tree Builder (https://www.geneious.com) and the randomized axelerated maximum likelihood plugin.

### Virulome and Resistome Analyses and Susceptibility Testing

We included virulence and resistance genes of *L. monocytogenes* as target loci in SeqSphere task templates, as previously described (*17*,*29*). We extracted targets from assembled contigs by using SeqSphere and considered alleles present when identity was >90% and the query sequence aligned >99% with the reference sequence.

We performed antimicrobial drug susceptibility testing by using a microdilution assay in a 96-well plate format adapted from a study by Noll et al. (*30*). All susceptibility testing was performed in accordance with European Committee on Antimicrobial Susceptibility Testing guidelines in Mueller Hinton fastidious (MH-F) broth (*31*). 

We spread *L. monocytogenes* isolates on BHI agar plates and placed 6 mm cellulose discs loaded with 10 µL of a 10 mg/mL aqueous benzalkonium chloride solution on top of the agar plate. We incubated plates at 37°C overnight, then determined growth inhibition zone diameters. We used the Student *t*-test to assess statistical significance.

### Case-Control Study

We defined outbreak cases as patients reported to public health authorities with disease onset during August 2018–June 2019 and isolation of *L. monocytogenes* from normally sterile body fluids and confirmation by cgMLST and SNP analysis. *L. monocytogenes* isolates were sent to the Robert Koch Institute (Wernigerode, Germany) and notification and typing data were merged for investigation. After the outbreak was identified, patients were interviewed by using a standardized questionnaire on food consumption during the 2 weeks before illness onset, general eating habits, and food purchasing behaviors. These data identified 40 food items for inclusion in the case-control study. 

We collaborated with a survey institute to contact and interview case-controls. We frequency matched case-controls to case-patients for age, gender, and federal state of residence. We considered food items with p<0.05 consumed by >50% of participants for multivariable analysis. We used a stepwise-backward approach for model formation to consecutively exclude food items that were no longer significantly associated from the multivariable model until only significantly associated foods and their confounders remained. We determined risk measures, including odds, univariable, and multivariable ratios, in the statistical analysis. 

## Results

### Molecular Surveillance

**Figure 1:**
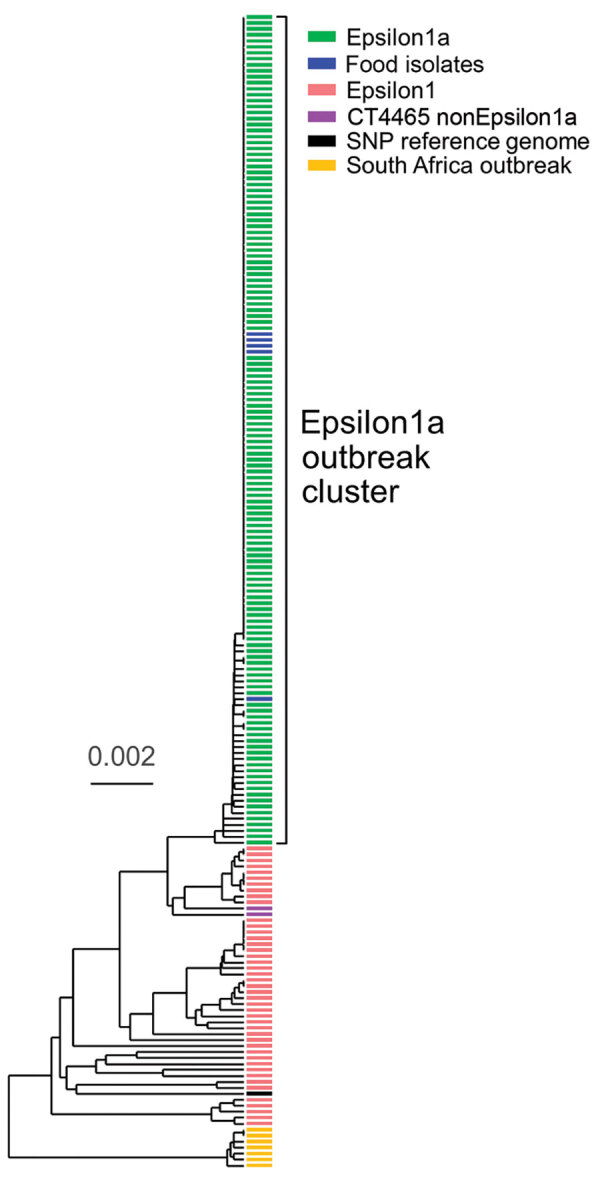
Phylogenic tree constructed by using unweighted pair group method with arithmetic mean and core genome multilocus sequence typing data of *Listeria monocytogenes* isolates from a large listeriosis outbreak, Germany. Green indicates clinical isolates of Epsilon1a subcluster; blue indicates food isolates of Epsilon1a subcluster; pink indicates isolates from the Epsilon1 cluster; violet indicates 2 complex type 4465 isolates not belonging to Epsilon1a from earlier listeriosis cases in July 2017 and June 2018; yellow indicates isolates from a listeriosis outbreak in South Africa (*11*); black indicates reference strain 10-092876-0769 LM12 used for SNP calling (Appendix Figure 2). Scale bar indicates allelic substitutions per site. SNP, single-nucleotide polymorphism.

The binational German–Austrian Consultant Laboratory for *L. monocytogenes* collects and sequences genomes of isolates from approximately two thirds of all mandatorily notified listeriosis cases in Germany; 699 cases were notified in 2018 and 593 in 2019. A phylogenetically diverse cluster, designated Epsilon1, was identified by using cgMLST. Epsilon1 included 46 PCR serogroup IVb isolates belonging to ST6 and CT90, CT2981, CT3803, CT3805, CT3806, CT3921, CT4083, CT4465, CT6236, CT6331, CT7353, and CT7451, all of which had specific allelic profiles within a CT threshold of <10 different alleles (*17*,*28*). The Epsilon1 cluster included isolates collected from all over Germany during 2011–2019 with no apparent geographic concentration. Allelic distances between isolates varied from 0–25 (median 11). In autumn 2018, a sudden increase of CT4465 and CT7353 isolates belonging to the Epsilon1 cluster was detected. Furthermore, the number of listeriosis cases reported in calendar weeks 34–43, 46, 48, and 50 exceeded the median of the 5 previous years (Appendix Figure 1). To identify the outbreak clone among all incoming serogroup IVb isolates, we developed a clone-specific PCR (Appendix). Altogether, 134 clinical CT4465 and CT7353 isolates were collected during August 2018–April 2019. These isolates formed a remarkably homogenous cluster with 0–5 (median 0) different cgMLST alleles (Figure 1). In contrast, 2 CT4465 isolates collected earlier, in July 2017 and June 2018, differed in 9–15 alleles (Figure 1).

We mapped raw sequence reads of all Epsilon1 strains against the 10-092876-0769 LM12 genome, the most closely related complete genome available. SNP calling separated the Epsilon1 cluster into several subclusters, but all CT4465 and CT7353 isolates collected from August 2018 onwards formed a single cluster (Appendix Figure 2). This subcluster was named Epsilon1a, and SNP distances in this cluster ranged from 0–3 SNPs (median 0). The 2 earlier CT4465 isolates were separated from the Epsilon1a cluster by 6–10 SNPs difference (median 8). Thus, SNP calling supported detection of a cluster of closely related CT4465 and CT7353 strains. Of note, only 21–29 cgMLST alleles (median 26) and 8–12 SNPs (median 8) differed between the Epsilon1a clone and the outbreak strain from South Africa (*11*), CT5886 (Figure 1; Appendix Figure 2).

### Case Cohort

**Figure 2:**
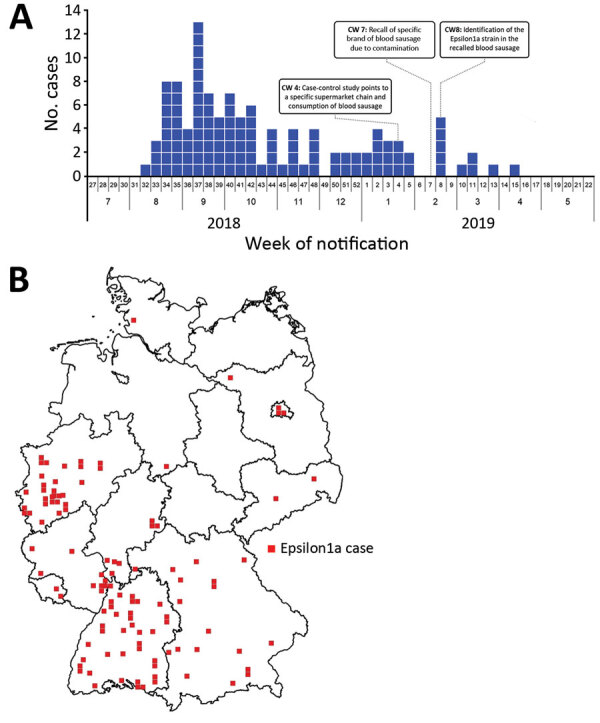
Spatial and temporal distribution of cases during a large listeriosis outbreak, Germany. A) Number of *Listeria monocytogenes* isolates from subcluster Epsilon1a received by the consulting laboratory per week during the outbreak. B) Geographic distribution of laboratory-confirmed Epsilon1a cases in Germany during the outbreak. CW, calendar week.

We collected 134 isolates from 112 patients who met the case definition. Initial cases were reported in August 2018, and the outbreak peaked in September 2018 (Figure 2, panel A); the last notified case was in April 2019. Cases occurred in 11/16 federal states in Germany; most cases occurred in western and southern Germany (Figure 2, panel B). This outbreak and the assembled genome of 1 representative isolate (isolate no. 18-04540) were shared via the Epidemic Intelligence Information System platform of the European Centre for Disease Prevention and Control on October 23, 2018 (UI-516, https://www.ecdc.europa.eu). France, the only other country involved, reported an Epsilon1a listeriosis case in a patient who had traveled to and purchased food in Germany. Sequence data of isolate 18-04540 was submitted to the European Nucleotide Archive (https://www.ebi.ac.uk/ena; accession no. SAMEA5041142). However, the closest related isolate available at the National Center for Biotechnology Information (https://www.ncbi.nlm.nih.gov) pathogen detection pipeline was a 2016 clinical isolate from the Netherlands with an SNP distance of 12, which is clearly above the SNP distances observed in the Epsilon1a cluster.

One (0.9%) case-patient was pregnant, but the gestational age and health outcome of her newborn were not reported. The remaining 111 case-patients were 53–98 (median 79) years of age; 66 (59%) were men, 45 (41%) were women. Seven (6.3%) case-patients died, 2 of whom had listeriosis as the primary cause of death. The age distribution was not noticeably different from other notified listeriosis outbreaks. Of the 134 Epsilon1a isolates, 99 were from blood samples, 13 from cerebrospinal fluid, and 1 each from lymph nodes, ascites, sputum, pleura, joints, abscesses, or a superficial wound (Appendix Table 1). The isolation source was not reported for the remaining 15 isolates.

### Properties of the Outbreak Clone

Virulome analysis revealed the presence of *Listeria* pathogenicity island 1 (LIPI-1) in all Epsilon1a outbreak isolates and detected the complete listeriolysin S-encoding LIPI-3 in 64% (Appendix Figure 3). However, we did not detect LIPI-4, which encodes a putative phosphotransferase system associated with neurolisteriosis (*32*). Epsilon1a clones carried the same complement of internalin genes as other serogroup IVb strains (Appendix Figure 3). 

**Figure 3:**
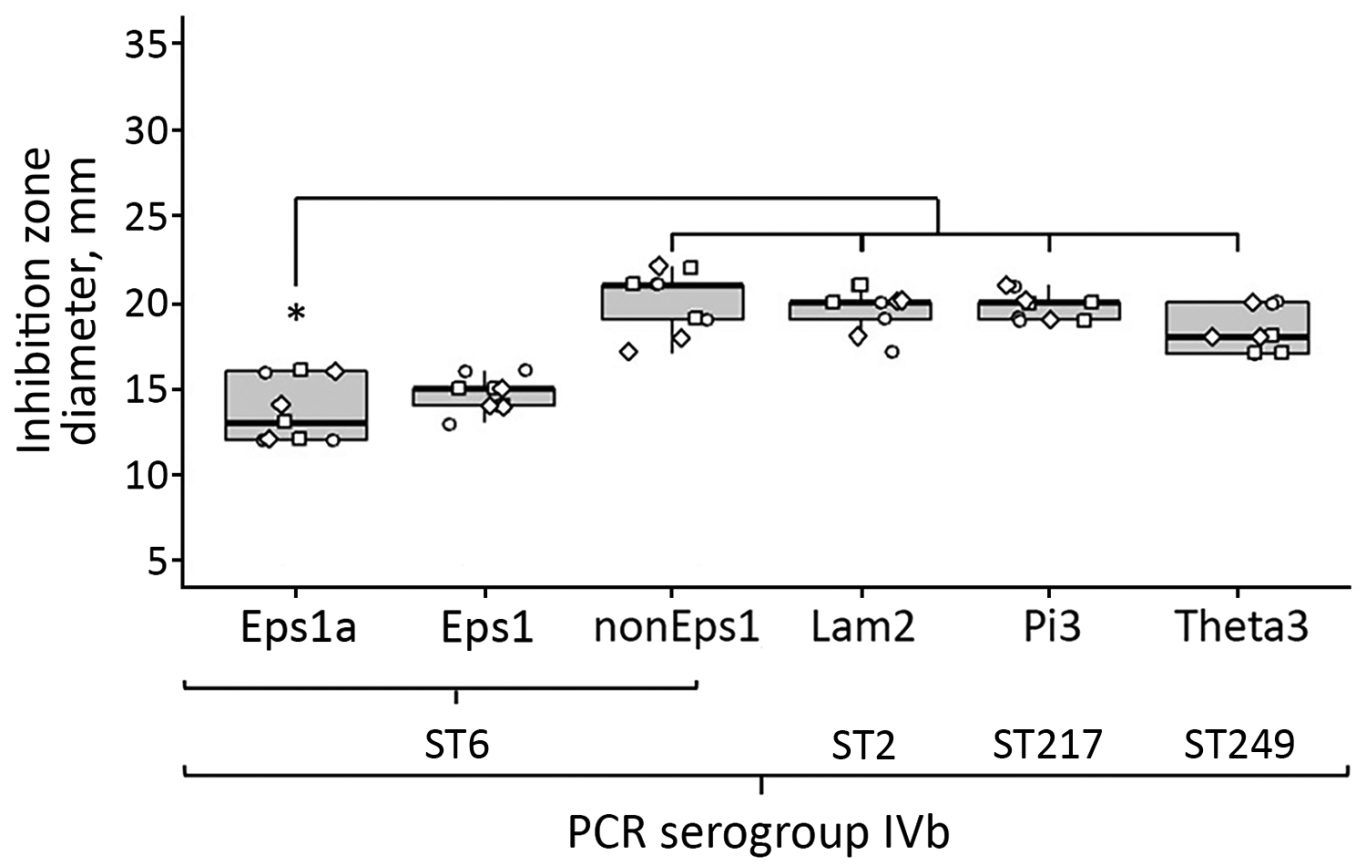
Tolerance of isolates of *Listeria monocytogenes* from subcluster Epsilon1a in Germany to benzalkonium chloride. Three representative isolates from human listeriosis clusters Epsilon1a, Epsilon1, and distinct listeriosis clusters Lambda2 (ST2, CT2402), Pi3 (ST217, CT5744), or Theta3 (ST249, CT4449) were tested for resistance to benzalkonium chloride by disc diffusion, along with 3 representative ST6 isolates, not belonging to Epsilon1. Epsilon1a and Epsilon1 isolates showed increased resistance to benzalkonium chloride. Circles, squares, and diamonds represent results of 3 independent replicates for 3 isolates per group. Asterisk indicates statistically significant differences to Epsilon1a (p<0.01) calculated by using the Student *t*-test. CT, complex type; Eps1, Epsilon1; Eps1a, Epsilon1a; Lam2, Lambda2; nonEps1, nonEpsilon1; ST, sequence type.

Susceptibility testing revealed sensitivity toward most clinically relevant antimicrobial drugs, but all tested isolates were fully resistant to ceftriaxone and daptomycin (Appendix Table 2), which is consistent with the intrinsic resistance of *L. monocytogenes* and the absence of additional resistance determinants, as suggested by the resistome approach (Appendix Figure 4). Further resistome analysis demonstrated the prevalence of the *emrC* gene, which is associated with benzalkonium chloride tolerance (Appendix Figure 4). In full agreement with this observation, Epsilon1a and Epsilon1 isolates demonstrated increased tolerance to benzalkonium chloride compared with ST6 or serogroup IVb isolates from other outbreak clusters (Figure 3).

### Identification the Outbreak Vehicle

Our case-control study included 41 case-patients and 155 controls. A total of 40/41 (98%) case-patients reported that they purchased food in a single specific supermarket chain, compared with 99/154 controls (64.3%; odds ratio [OR] 22.5, 95% CI 2.9–174.9; p = 0.003). No other supermarket chains were associated with outbreak cases, and we only included case-patients and controls that had purchased food from the specific supermarket chain in further analyses. In the fourth calendar week of 2019, we detected a strong association between cases and consumption of minced meat (OR 42.4, 95% CI 4.3–415.4; p = 0.001) and blood sausage (OR 23.1, 95% CI 4.3–123.5; p<0.001; Table). Among case-patients, 90% reported consuming minced meat and 80% reported consuming blood sausage compared with 23% of controls who consumed minced meat and 45% who consumed blood sausage. None of the case-patients were vegetarians.

**Table:** Results of multivariable analysis of risk for infection by food consumption from a case-control study during listeriosis outbreak, Germany 2018–2019*

Food item	Odds ratio (95% CI)†	p value
Minced meat	42.4 (4.3–415.1)	0.001
Blood sausage	23.1 (4.3–123.5)	<0.001
Cold cuts, including roast pork and Kassler	15.4 (2.9–82.1)	0.001
Edamer cheese	7.3 (1.6–32.8)	0.009
Smoked ham‡	0.06 (0.0–0.4)	0.003
Hard cheese‡	0.2 (0.0–0.9)	0.038

*Includes 40/41 cases and 99/155 controls that confirmed shopping at the implicated supermarket chain. †Adjusted for age, gender, and residence in northern or southern region. **‡Smoked ham and hard cheese are confounders for cold cuts and Edamer cheese.**

To perform risk-oriented screening, food samples were collected in supermarkets and the households of some patients, according to the results of the epidemiologic investigations. In 1 case, *L. monocytogenes* was detected in 3 open samples from a patient’s refrigerator. Among these, sliced blood sausage purchased at the implicated supermarket chain showed the highest contamination (>3 × 10^6^ CFU/g). This finding led to another round of intensified screening of prepackaged blood sausage. In calendar week 7 of 2019, *L. monocytogenes* was found in an original sealed package of sliced blood sausage (<10 CFU/g) and in a second sample of blood sausage from the same manufacturer. In total, 5 isolates from patients’ household food items and from blood sausage samples grouped with clinical Epsilon1a isolates after cgMLST (0–3 different alleles, median = 0) and SNP calling (0–2 SNPs, median = 0) (Figure 1; Appendix Figure 2). 

The blood sausage was produced by a large meat and sausage manufacturer in Germany and sold in many parts of the country. The product was withdrawn from the market on February 12, 2019. The last clinical Epsilon1a isolate was collected on April 18, 2019. In contrast, Epsilon1 isolates not belonging to the Epsilon1a cluster caused disease even after the end of the Epsilon1a outbreak. The plant was cleaned and disinfected. Thereafter, *L. monocytogenes* was not detected from products or the production site among several hundred samples collected by food safety inspectors and ≈2,500 control samples collected by the manufacturer.

## Discussion

The Epsilon1a outbreak is the largest identified outbreak of listeriosis in Germany and one of the largest documented outbreaks of invasive listeriosis in Europe in >25 years. The last reported outbreak of invasive listeriosis of this size in Europe was during 1992–1993 when 247 patients were infected in France with a serotype IVb clone from contaminated pork tongue in aspic (*33*). Cantaloupe was the vehicle in the large outbreak in the United States in 2011 that was caused by 5 different clones (*10*). In contrast, the single clone that caused a large outbreak in South Africa showed strong clonality and the genomes of 326 isolates differed in <4 cgMLST alleles (*11*). Likewise, the Epsilon1a outbreak was caused by a single clone, and we observed high clonality among isolates. The mutation rate in the natural *L. monocytogenes* population is 2.6 × 10^−7^ substitutions per site per year (*29*). On average, 1 SNP/year can be expected for *L. monocytogenes* strains under natural conditions. The high clonality of Epsilon1a could imply that the outbreak clone only persisted in the production facility and did not undergo rapid multiplication.

Purchases in a particular supermarket chain and consumption of blood sausage were strongly associated with listeriosis in the case-control study, and the outbreak clone was identified in blood sausage samples from a patient’s household and from the implicated supermarket chain. Blood sausage is heat-treated during production, so contamination likely occurred after production, possibly during slicing or packaging. The shelf-life of sliced blood sausage is several days to a few weeks (*34*) and the amount of *L. monocytogenes* found in unopened blood sausage samples was below the limit of 100 CFU/g. Storage beyond the anticipated shelf-life or insufficient refrigeration might have allowed *L. monocytogenes* to multiply inside the vehicle, which would only be prevented by a zero-tolerance policy. 

Typically, pregnancy-related listeriosis accounts for ≈7% of all listeriosis cases (*35*). However, in this outbreak only 1/112 (0.8%) cases was in a pregnant woman. Official recommendations for pregnant women to apply special hygiene practices to sliced sausage products likely had an effect (*36*).

Analysis of the Epsilon1a genome has yielded some insights into the infectivity of this ST6 clone. *L. monocytogenes* ST6 clones were first isolated in 1990 (*37*), have caused various outbreaks in the past, including the large outbreak in South Africa (*11*), and are associated with increased rates of meningitis (*38*). The Epsilon1a clone and the outbreak strain from South Africa are closely related. Thus, 2 descendants of the same historic *L. monocytogenes* ancestor have spread globally and contaminated food production facilities on 2 different continents. The Epsilon1a clone carried the *emrC* gene, which presumably caused its increased tolerance to benzalkonium chloride (*39*). Europe banned use of benzalkonium chloride as a disinfectant in 2016 (*40*), but its past use might have selected tolerant strains.

The identification of this outbreak and its vehicle resulted from an efficient collaboration between public health and food safety authorities in Germany. Several requirements had to be met for successful outbreak clarification: development of a mandatory notification system for systematic patient interviews and an efficient questionnaire to generate hypotheses on possible food sources; implementation of a WGS-based molecular surveillance program for reliable identification of outbreak clusters by public health authorities; systematic collection of food isolates from internal controls and on-demand investigations and use of a harmonized WGS-based subtyping methodology by food safety authorities; and a continuous exchange of information on outbreak clusters between the institutions involved. These prerequisites have identified the causative food vehicles for 5 of 6 large listeriosis clusters that occurred in Germany during 2014–2019, which likely would not have been possible before use of WGS in outbreak investigations. However, introduction of routine interviews of listeriosis patients, regardless of outbreaks, probably could further accelerate identification of outbreak vehicles. In our opinion, the Epsilon1a outbreak demonstrates how WGS-based pathogen surveillance combined with efficient interventions of the involved stakeholders can improve management and prevention of foodborne infectious diseases.

Appendix Additional information on large nationwide outbreak of invasive listeriosis associated with blood sausage consumption, Germany, 2018–2019.
