# Supplementary material for: Large Nationwide Outbreak of Invasive Listeriosis Associated with Blood Sausage, Germany, 2018–2019
Source: Emerg Infect Dis. 2020 Jul;26(7):1456–64. doi: 10.3201/eid2607.200225 (PMC7323541; doi:10.3201/eid2607.200225)
Supplement: Appendix — Additional information on large nationwide outbreak of invasive listeriosis associated with blood sausage consumption, Germany, 2018–2019. [file 20-0225-Techapp-s1.pdf]

Article DOI: https://doi.org/10.3201/eid2607.200225

Large Nationwide Outbreak of Invasive Listeriosis Associated with Blood Sausage, Germany, 2018–2019

Appendix

Additional Methods

Diagnostic PCR for Identification of the Outbreak Clone

During this investigation, the median time from arrival of a sample in the consultant laboratory until genome sequencing results was 34.5 days. Therefore, we searched for DNA regions specific for Epsilon1a isolates to identify Epsilon1a clones by PCR and prioritize samples for genome sequencing and initiate patient interviews as soon as possible. For this purpose, the contigs of 3 Epsilon1a isolates (target) and of 50 nonEpsilon1a isolates of serogroup IVb (nontarget) were analyzed by the RUCS 1.0 algorithm, designed to identify primer pairs for unique core sequences in a target genome dataset and absent in a set of nontarget genomes (*1*). This approach identified a 262 bp fragment specific for the chosen target genomes that could be amplified by using the primers Eps-1-fw (AGTCGTCTTTAGTGCGCTGAA) and Eps-1-rev (TAGGTCTGTTGATGGCACCAC). The applicability of this primer set was tested by using 16 genome sequences of Epsilon1a and 12 of nonEpsilon1a serogroup IVb clones. Experimentally determined sensitivity of the PCR system was 94% and specificity was 75%. The 262 bp fragment is part of an open reading frame encoding a phage tail length tape measure protein, which was detected in 119/134 (89%) Epsilon1a strains according to genomic data. In contrast, among 662 analyzed serogroup IVb genomes, this phage tail open reading frame was found in only 20 (3%) ST6 genomes that did not belong to the Epsilon1a group. Thus, the Eps1a PCR was used to identify possible Epsilon1a isolates among the incoming serogroup IVb isolates to prioritize them for WGS. Of the 67 serogroup IVb isolates that had been tested by the Eps1a PCR during the outbreak, only 11 (16%) were false-positive.

References

1. Thomsen MCF, Hasman H, Westh H, Kaya H, Lund O. RUCS: rapid identification of PCR primers for unique core sequences. Bioinformatics. 2017;33:3917–21. PubMed https://doi.org/10.1093/bioinformatics/btx526

2. Kropac AC, Eshwar AK, Stephan R, Tasara T. New insights on the role of the pLMST6 plasmid in *Listeria monocytogenes* biocide tolerance and virulence. Front Microbiol. 2019;10:1538. PubMed https://doi.org/10.3389/fmicb.2019.01538

Appendix Table 1. *Listeria monocytogenes* isolates included from a large listeriosis outbreak, Germany, 2018–2019*

Isolate ID	Sample accession no.	Secondary accession no.	Study number	Type	Source	Cluster	Complex type
11–04869	SAMEA104485064	ERS2103006	PRJEB24496	Human	Blood	Epsilon1	90
16–00332	SAMEA104485223	ERS2103165	PRJEB24496	Human	Blood	Epsilon1	90
16–00478	SAMEA104485231	ERS2103173	PRJEB24496	Human	Blood	Epsilon1	90
16–00634	SAMEA104485236	ERS2103178	PRJEB24496	Human	CSF	Epsilon1	90
16–00830	SAMEA5770458	ERS3574002	PRJEB33238	Human	Blood	Epsilon1	90
16–00831	SAMEA104485244	ERS2103186	PRJEB24496	Human	Uterus	Epsilon1	90
16–00955	SAMEA104485248	ERS2103190	PRJEB24496	Human	Blood	Epsilon1	90
16–01401	SAMEA104485262	ERS2103204	PRJEB24496	Human	Blood	Epsilon1	90
16–01909	SAMEA104485285	ERS2103227	PRJEB24496	Human	Blood	Epsilon1	90
16–01911	SAMEA104485286	ERS2103228	PRJEB24496	Human	Blood	Epsilon1	90
16–02052	SAMEA104485291	ERS2103233	PRJEB24496	Human	Unknown	Epsilon1	3803
16–02281	SAMEA104485298	ERS2103240	PRJEB24496	Human	Blood	Epsilon1	3805
16–02328	SAMEA104485301	ERS2103243	PRJEB24496	Human	Unknown	Epsilon1	3806
16–02495	SAMEA104485307	ERS2103249	PRJEB24496	Human	Unknown	Epsilon1	90
16–02497	SAMEA104485309	ERS2103251	PRJEB24496	Human	Blood	Epsilon1	2981
16–02650	SAMEA104485313	ERS2103255	PRJEB24496	Human	Blood	Epsilon1	3921
16–03183	SAMEA104485347	ERS2103289	PRJEB24496	Human	CSF	Epsilon1	4083
16–04063	SAMEA5770459	ERS3574003	PRJEB33238	Human	Blood	Epsilon1	90
16–04236	SAMEA104485396	ERS2103338	PRJEB24496	Human	Blood	Epsilon1	90
16–04386	SAMEA5770460	ERS3574004	PRJEB33238	Human	Blood	Epsilon1	90
16–04399	SAMEA104485408	ERS2103350	PRJEB24496	Human	Blood	Epsilon1	90
16–04799	SAMEA104485422	ERS2103364	PRJEB24496	Human	Ascites	Epsilon1	90
16–04800	SAMEA104485423	ERS2103365	PRJEB24496	Human	Blood	Epsilon1	90
16–05014	SAMEA104485430	ERS2103372	PRJEB24496	Human	Ascites	Epsilon1	90
17–00454	SAMEA104485458	ERS2103400	PRJEB24496	Human	Blood	Epsilon1	4083
17–00659	SAMEA5769034	ERS3572580	PRJEB33238	Human	Blood	Epsilon1	2981
17–01077	SAMEA5769035	ERS3572581	PRJEB33238	Human	Blood	Epsilon1	90
17–03140	SAMEA5769036	ERS3572582	PRJEB33238	Human	Blood	Epsilon1	4465
17–05508	SAMEA5769037	ERS3572583	PRJEB33238	Human	Blood	Epsilon1	2981
17–06068	SAMEA5769038	ERS3572584	PRJEB33238	Human	Blood	Epsilon1	90
17–06319	SAMEA5769039	ERS3572585	PRJEB33238	Human	CSF	Epsilon1	90
17–06904	SAMEA5769040	ERS3572586	PRJEB33238	Human	Blood	Epsilon1	90
18–00080	SAMEA5769102	ERS3572648	PRJEB33238	Human	CSF	Epsilon1	90
18–00304	SAMEA5769041	ERS3572587	PRJEB33238	Human	Blood	Epsilon1	6236
18–00445	SAMEA5769103	ERS3572649	PRJEB33238	Human	Unknown	Epsilon1	6331
18–01855	SAMEA5769042	ERS3572588	PRJEB33238	Human	Blood	Epsilon1	90
18–02683	SAMEA5769104	ERS3572650	PRJEB33238	Human	Blood	Epsilon1	4465
18–02987	SAMEA5769105	ERS3572651	PRJEB33238	Human	GS	Epsilon1	90
18–03576	SAMEA5769106	ERS3572652	PRJEB33238	Human	Unknown	Epsilon1	2981
18–03577	SAMEA5769107	ERS3572653	PRJEB33238	Human	GS	Epsilon1	2981
18–04116	SAMEA5769108	ERS3572654	PRJEB33238	Human	Blood	Epsilon1a	4465
18–04317	SAMEA5769109	ERS3572655	PRJEB33238	Human	CSF	Epsilon1a	7353
18–04364	SAMEA5769110	ERS3572656	PRJEB33238	Human	Blood	Epsilon1a	4465
18–04365	SAMEA5769043	ERS3572589	PRJEB33238	Human	Blood	Epsilon1a	4465
18–04414	SAMEA5769111	ERS3572657	PRJEB33238	Human	CSF	Epsilon1a	7353
18–04434	SAMEA5769112	ERS3572658	PRJEB33238	Human	Blood	Epsilon1a	4465
18–04472	SAMEA5769113	ERS3572659	PRJEB33238	Human	Blood	Epsilon1	6331
18–04499	SAMEA5769114	ERS3572660	PRJEB33238	Human	Blood	Epsilon1a	4465

Isolate ID	Sample accession no.	Secondary accession no.	Study number	Type	Source	Cluster	Complex type
18–04500	SAMEA5769115	ERS3572661	PRJEB33238	Human	Blood	Epsilon1a	4465
18–04539	SAMEA5769116	ERS3572662	PRJEB33238	Human	Blood	Epsilon1a	4465
18–04540	SAMEA5041142	ERS2852884	PRJEB29295	Human	Blood	Epsilon1a	4465
18–04543	SAMEA5769044	ERS3572590	PRJEB33238	Human	Blood	Epsilon1a	4465
18–04581	SAMEA5769045	ERS3572591	PRJEB33238	Human	Blood	Epsilon1a	4465
18–04652	SAMEA5769046	ERS3572592	PRJEB33238	Human	Blood	Epsilon1a	4465
18–04653	SAMEA5769047	ERS3572593	PRJEB33238	Human	Blood	Epsilon1a	7353
18–04654	SAMEA5769048	ERS3572594	PRJEB33238	Human	Blood	Epsilon1a	4465
18–04655	SAMEA5769049	ERS3572595	PRJEB33238	Human	Blood	Epsilon1a	4465
18–04657	SAMEA5769050	ERS3572596	PRJEB33238	Human	Blood	Epsilon1a	7353
18–04772	SAMEA5769051	ERS3572597	PRJEB33238	Human	Blood	Epsilon1a	4465
18–04825	SAMEA5769052	ERS3572598	PRJEB33238	Human	Blood	Epsilon1a	7353
18–04826	SAMEA5769053	ERS3572599	PRJEB33238	Human	Blood	Epsilon1a	4465
18–04827	SAMEA5769117	ERS3572663	PRJEB33238	Human	Blood	Epsilon1a	7353
18–04850	SAMEA5769118	ERS3572664	PRJEB33238	Human	Blood	Epsilon1a	4465
18–04852	SAMEA5769119	ERS3572665	PRJEB33238	Human	Blood	Epsilon1a	4465
18–04897	SAMEA5769120	ERS3572666	PRJEB33238	Human	Blood	Epsilon1a	7353
18–04898	SAMEA5769121	ERS3572667	PRJEB33238	Human	Blood	Epsilon1a	4465
18–04954	SAMEA5769122	ERS3572668	PRJEB33238	Human	Blood	Epsilon1a	4465
18–04955	SAMEA5769123	ERS3572669	PRJEB33238	Human	Blood	Epsilon1a	4465
18–05034	SAMEA5769124	ERS3572670	PRJEB33238	Human	Blood	Epsilon1	7451
18–05035	SAMEA5769125	ERS3572671	PRJEB33238	Human	Blood	Epsilon1a	7353
18–05038	SAMEA5769126	ERS3572672	PRJEB33238	Human	Blood	Epsilon1a	4465
18–05084	SAMEA5769127	ERS3572673	PRJEB33238	Human	Blood	Epsilon1a	4465
18–05142	SAMEA5769128	ERS3572674	PRJEB33238	Human	Unknown	Epsilon1a	4465
18–05143	SAMEA5769129	ERS3572675	PRJEB33238	Human	Unknown	Epsilon1a	7353
18–05144	SAMEA5769130	ERS3572676	PRJEB33238	Human	Blood	Epsilon1a	4465
18–05199	SAMEA5769131	ERS3572677	PRJEB33238	Human	Blood	Epsilon1a	4465
18–05201	SAMEA5769132	ERS3572678	PRJEB33238	Human	Blood	Epsilon1a	4465
18–05202	SAMEA5769133	ERS3572679	PRJEB33238	Human	Blood	Epsilon1a	4465
18–05203	SAMEA5769134	ERS3572680	PRJEB33238	Human	Blood	Epsilon1a	4465
18–05237	SAMEA5769135	ERS3572681	PRJEB33238	Human	Blood	Epsilon1a	4465
18–05327	SAMEA5769136	ERS3572682	PRJEB33238	Human	Blood	Epsilon1a	4465
18–05328	SAMEA5769137	ERS3572683	PRJEB33238	Human	Blood	Epsilon1a	4465
18–05329	SAMEA5769138	ERS3572684	PRJEB33238	Human	Blood	Epsilon1a	4465
18–05393	SAMEA5769139	ERS3572685	PRJEB33238	Human	CSF	Epsilon1a	4465
18–05394	SAMEA5769140	ERS3572686	PRJEB33238	Human	Blood	Epsilon1a	4465
18–05396	SAMEA5769141	ERS3572687	PRJEB33238	Human	Blood	Epsilon1a	7353
18–05398	SAMEA5769054	ERS3572600	PRJEB33238	Human	Blood	Epsilon1a	7353
18–05449	SAMEA5769142	ERS3572688	PRJEB33238	Human	Blood	Epsilon1a	4465
18–05450	SAMEA5769143	ERS3572689	PRJEB33238	Human	Blood	Epsilon1a	4465
18–05496	SAMEA5769144	ERS3572690	PRJEB33238	Human	Blood	Epsilon1a	4465
18–05542	SAMEA5769145	ERS3572691	PRJEB33238	Human	CSF	Epsilon1a	4465
18–05544	SAMEA5769146	ERS3572692	PRJEB33238	Human	Blood	Epsilon1a	4465
18–05558	SAMEA5769147	ERS3572693	PRJEB33238	Human	Blood	Epsilon1a	4465
18–05655	SAMEA5769148	ERS3572694	PRJEB33238	Human	Blood	Epsilon1a	4465
18–05657	SAMEA5769149	ERS3572695	PRJEB33238	Human	CSF	Epsilon1a	4465
18–05658	SAMEA5769150	ERS3572696	PRJEB33238	Human	Blood	Epsilon1a	4465
18–05660	SAMEA5769151	ERS3572697	PRJEB33238	Human	Blood	Epsilon1a	4465
18–05714	SAMEA5769152	ERS3572698	PRJEB33238	Human	Blood	Epsilon1a	4465
18–05726	SAMEA5769153	ERS3572699	PRJEB33238	Human	Blood	Epsilon1a	7353
18–05748	SAMEA5769154	ERS3572700	PRJEB33238	Human	Blood	Epsilon1a	4465
18–05767	SAMEA5769155	ERS3572701	PRJEB33238	Human	Blood	Epsilon1a	4465
18–05768	SAMEA5769156	ERS3572702	PRJEB33238	Human	CSF	Epsilon1a	4465
18–05836	SAMEA5769157	ERS3572703	PRJEB33238	Human	Unknown	Epsilon1a	7353
18–05837	SAMEA5769055	ERS3572601	PRJEB33238	Human	Blood	Epsilon1a	7353
18–05951	SAMEA5769158	ERS3572704	PRJEB33238	Human	Blood	Epsilon1a	4465
18–05970	SAMEA5769159	ERS3572705	PRJEB33238	Human	CSF	Epsilon1a	4465
18–06023	SAMEA5769056	ERS3572602	PRJEB33238	Human	Unknown	Epsilon1a	7353
18–06024	SAMEA5769057	ERS3572603	PRJEB33238	Human	Ascites	Epsilon1a	4465
18–06035	SAMEA5769058	ERS3572604	PRJEB33238	Human	Blood	Epsilon1a	4465
18–06036	SAMEA5769059	ERS3572605	PRJEB33238	Human	Blood	Epsilon1a	4465
18–06121	SAMEA5769060	ERS3572606	PRJEB33238	Human	Blood	Epsilon1a	4465
18–06126	SAMEA5769061	ERS3572607	PRJEB33238	Human	Blood	Epsilon1a	4465
18–06127	SAMEA5769062	ERS3572608	PRJEB33238	Human	CSF	Epsilon1a	4465
18–06128	SAMEA5769063	ERS3572609	PRJEB33238	Human	Blood	Epsilon1a	4465
18–06129	SAMEA5769064	ERS3572610	PRJEB33238	Human	Blood	Epsilon1a	4465
18–06130	SAMEA5769065	ERS3572611	PRJEB33238	Human	Blood	Epsilon1a	7353

Isolate ID	Sample accession no.	Secondary accession no.	Study number	Type	Source	Cluster	Complex type
18–06131	SAMEA5769066	ERS3572612	PRJEB33238	Human	CSF	Epsilon1a	4465
18–06138	SAMEA5769067	ERS3572613	PRJEB33238	Human	Blood	Epsilon1a	4465
18–06170	SAMEA5769160	ERS3572706	PRJEB33238	Human	Unknown	Epsilon1a	4465
18–06263	SAMEA5769161	ERS3572707	PRJEB33238	Human	Blood	Epsilon1a	4465
18–06331	SAMEA5769162	ERS3572708	PRJEB33238	Human	Blood	Epsilon1a	4465
18–06438	SAMEA5769163	ERS3572709	PRJEB33238	Human	Unknown	Epsilon1a	4465
18–06540	SAMEA5769164	ERS3572710	PRJEB33238	Human	Blood	Epsilon1a	4465
18–06541	SAMEA5769165	ERS3572711	PRJEB33238	Human	CSF	Epsilon1a	4465
18–06646	SAMEA5769166	ERS3572712	PRJEB33238	Human	CSF	Epsilon1a	4465
18–06680	SAMEA5769167	ERS3572713	PRJEB33238	Human	Blood	Epsilon1a	4465
18–06776	SAMEA5769168	ERS3572714	PRJEB33238	Human	Blood	Epsilon1a	4465
18–06820	SAMEA5769169	ERS3572715	PRJEB33238	Human	Unknown	Epsilon1a	7353
18–06916	SAMEA5769170	ERS3572716	PRJEB33238	Human	Blood	Epsilon1a	4465
18–06954	SAMEA5769068	ERS3572614	PRJEB33238	Human	Blood	Epsilon1a	7353
18–06955	SAMEA5769069	ERS3572615	PRJEB33238	Human	Blood	Epsilon1a	4465
18–07018	SAMEA5769070	ERS3572616	PRJEB33238	Human	Blood	Epsilon1a	4465
18–07037	SAMEA5769071	ERS3572617	PRJEB33238	Human	Blood	Epsilon1a	4465
18–07092	SAMEA5769072	ERS3572618	PRJEB33238	Human	Unknown	Epsilon1a	4465
18–07157	SAMEA5769073	ERS3572619	PRJEB33238	Human	Blood	Epsilon1a	4465
18–07158	SAMEA5769074	ERS3572620	PRJEB33238	Human	Blood	Epsilon1a	4465
18–07267	SAMEA5769171	ERS3572717	PRJEB33238	Human	Blood	Epsilon1a	4465
18–07300	SAMEA5769172	ERS3572718	PRJEB33238	Human	Unknown	Epsilon1a	7353
19–00076	SAMEA5769173	ERS3572719	PRJEB33238	Human	Blood	Epsilon1a	4465
19–00080	SAMEA5769174	ERS3572720	PRJEB33238	Human	Blood	Epsilon1	90
19–00082	SAMEA5769175	ERS3572721	PRJEB33238	Human	Unknown	Epsilon1a	4465
19–00149	SAMEA5769176	ERS3572722	PRJEB33238	Human	CSF	Epsilon1a	4465
19–00151	SAMEA5769177	ERS3572723	PRJEB33238	Human	Blood	Epsilon1a	4465
19–00179	SAMEA5769178	ERS3572724	PRJEB33238	Human	Unknown	Epsilon1a	4465
19–00191	SAMEA5769179	ERS3572725	PRJEB33238	Human	Blood	Epsilon1a	4465
19–00240	SAMEA5769180	ERS3572726	PRJEB33238	Human	Unknown	Epsilon1a	4465
19–00278	SAMEA5769181	ERS3572727	PRJEB33238	Human	Blood	Epsilon1a	4465
19–00281	SAMEA5769182	ERS3572728	PRJEB33238	Human	Blood	Epsilon1a	7353
19–00312	SAMEA5769075	ERS3572621	PRJEB33238	Human	Blood	Epsilon1a	7353
19–00347	SAMEA5769076	ERS3572622	PRJEB33238	Human	Blood	Epsilon1a	4465
19–00419	SAMEA5769077	ERS3572623	PRJEB33238	Human	Blood	Epsilon1a	4465
19–00444	SAMEA5769078	ERS3572624	PRJEB33238	Human	Blood	Epsilon1a	4465
19–00499	SAMEA5769079	ERS3572625	PRJEB33238	Human	Blood	Epsilon1a	4465
19–00500	SAMEA5769080	ERS3572626	PRJEB33238	Human	Blood	Epsilon1a	4465
19–00520	SAMEA5769081	ERS3572627	PRJEB33238	Human	Blood	Epsilon1a	4465
19–00549	SAMEA5769082	ERS3572628	PRJEB33238	Human	Blood	Epsilon1a	4465
19–00582	SAMEA5769083	ERS3572629	PRJEB33238	Human	Blood	Epsilon1a	7353
19–00609	SAMEA5769084	ERS3572630	PRJEB33238	Human	Unknown	Epsilon1a	7353
19–00973	SAMEA5769183	ERS3572729	PRJEB33238	Human	Unknown	Epsilon1a	4465
19–00974	SAMEA5769184	ERS3572730	PRJEB33238	Human	Blood	Epsilon1a	7353
19–00998	SAMEA5769185	ERS3572731	PRJEB33238	Human	Blood	Epsilon1a	4465
19–01023	SAMEA5769186	ERS3572732	PRJEB33238	Human	Blood	Epsilon1a	4465
19–01108	SAMEA5769187	ERS3572733	PRJEB33238	Human	Blood	Epsilon1a	4465
19–01166	SAMEA5769188	ERS3572734	PRJEB33238	Human	Placenta	Epsilon1	90
19–01173	SAMEA5769189	ERS3572735	PRJEB33238	Human	Blood	Epsilon1	90
19–01197	SAMEA5769190	ERS3572736	PRJEB33238	Human	Blood	Epsilon1a	4465
19–01319	SAMEA5769191	ERS3572737	PRJEB33238	Human	Synovia	Epsilon1a	4465
19–01387	SAMEA5769192	ERS3572738	PRJEB33238	Human	PF	Epsilon1a	4465
19–01604	SAMEA5769193	ERS3572739	PRJEB33238	Human	Wound	Epsilon1a	4465
19–01607	SAMEA5769194	ERS3572740	PRJEB33238	Human	BA	Epsilon1a	4465
19–01930	SAMEA5769195	ERS3572741	PRJEB33238	Human	Blood	Epsilon1a	4465
19–01961	SAMEA5769196	ERS3572742	PRJEB33238	Human	Blood	Epsilon1	90
19–02578	SAMEA5769085	ERS3572631	PRJEB33238	Human	Blood	Epsilon1a	7353
19–02579	SAMEA5769086	ERS3572632	PRJEB33238	Human	Blood	Epsilon1a	7353
19–02581	SAMEA5769087	ERS3572633	PRJEB33238	Human	Blood	Epsilon1a	4465
19–02587	SAMEA5769088	ERS3572634	PRJEB33238	Human	Blood	Epsilon1a	7353
19–02590	SAMEA5769089	ERS3572635	PRJEB33238	Human	CSF	Epsilon1a	4465
19–02598	SAMEA5769090	ERS3572636	PRJEB33238	Human	CSF	Epsilon1a	4465
19–02600	SAMEA5769091	ERS3572637	PRJEB33238	Human	Blood	Epsilon1a	4465
19-LI00135–0	SAMEA5769092	ERS3572638	PRJEB33238	Food	–	Epsilon1a	4465
19-LI00136–0	SAMEA5769093	ERS3572639	PRJEB33238	Food	–	Epsilon1a	4465
19-LI00137–0	SAMEA5769094	ERS3572640	PRJEB33238	Food	–	Epsilon1a	4465
19-LI00138–0	SAMEA5769095	ERS3572641	PRJEB33238	Food	–	Epsilon1a	4465
19-LI00175–0	SAMEA5769096	ERS3572642	PRJEB33238	Food	–	Epsilon1a	4465

Isolate ID	Sample accession no.	Secondary accession no.	Study number	Type	Source	Cluster	Complex type

*Isolates from nonsterile materials were not included in the outbreak description. BA, brain abscess; CSF, cerebrospinal fluid; GS, gynecological swab; PF, pleural fluid.

Appendix Table 2. Antimicrobial susceptibility in 79 isolates of *Listeria monocytogenes* from listeriosis outbreak cluster Epsilon1a, Germany*

Antimicrobial	MIC, mg/L (no. isolates)													
drug	0.003	0.06	0.125	0.25	0.5	1.0	2.0	4.0	8.0	16.0	32	64	128	>128
Ampicillin†	ND	–	–	S (29)	S (50)	I	–	–	–	ND	ND	ND	ND	ND
Benzylpenicillin†	ND	–	–	S (32)	S (43)	I	–	–	–	ND	ND	ND	ND	ND
Ceftriaxone‡	ND	ND	ND	ND	ND	I	I	R	R	R	R	R	R (6)	R (73)
Ciprofloxacin§	ND	ND	ND	–	–	I (67)	R (12)	–	–	ND	ND	ND	ND	ND
Daptomycin§	ND	ND	ND	ND	–	I	R (9)	R (68)	R (2)	–	ND	ND	ND	ND
Erythromycin†	ND	ND	ND	S (79)¶	–	I	–	–	–	ND	ND	ND	ND	ND
Gentamicin§	ND	ND	ND	ND	S (65)¶	I (14)	–	–	–	–	ND	ND	ND	ND
Linezolid#	ND	ND	ND	ND	–	–	I	I (79)	–	–	ND	ND	ND	ND
Meropenem**	ND	–	S (79)	I	–	–	–	–	–	ND	ND	ND	ND	ND
Rifampin††	ND	I (79)¶	I	I	I	–	–	ND	ND	ND	ND	ND	ND	ND
Tetracycline‡	ND	ND	ND	–	–	I (4)	I (75)	–	–	ND	ND	ND	ND	ND
Tigecycline‡‡	R	S (1)	S (77)	S (1)	I	–	–	–	ND	ND	ND	ND	ND	ND
Cotrimoxazole§§	S (79)¶	I	–	–	–	–	–	–	ND	ND	ND	ND	ND	ND
Vancomycin‡	ND	ND	ND	ND	ND	S (74)¶	I (5)	–	–	–	ND	ND	ND	ND

*All 79 strains were tested against 14 antimicrobial drugs. I, intermediate; R, resistant; S, susceptible; –, no isolates in this MIC category.
†EUCAST breakpoint 1.0 mg/mL for *Listeria monocytogenes*.
‡EUCAST breakpoint 2.0 mg/mL for *Streptococcus pneumoniae*.
§EUCAST breakpoint 1.0 mg/mL for *Staphylococcus aureus*.
¶No observable growth at the lowest tested concentration.
#EUCAST breakpoint 4.0 mg/mL for *Streptococcus pneumoniae*.
**EUCAST breakpoint 0.25 mg/mL for *Listeria monocytogenes*.
††EUCAST breakpoint 0.5 mg/mL for *Streptococcus pneumoniae*.
‡‡EUCAST breakpoint 0.5 mg/mL for *Staphylococcus aureus*.
§§Trimethoprim/sulfamethoxazole; EUCAST breakpoint 0.06 mg/mL for *Listeria monocytogenes*.

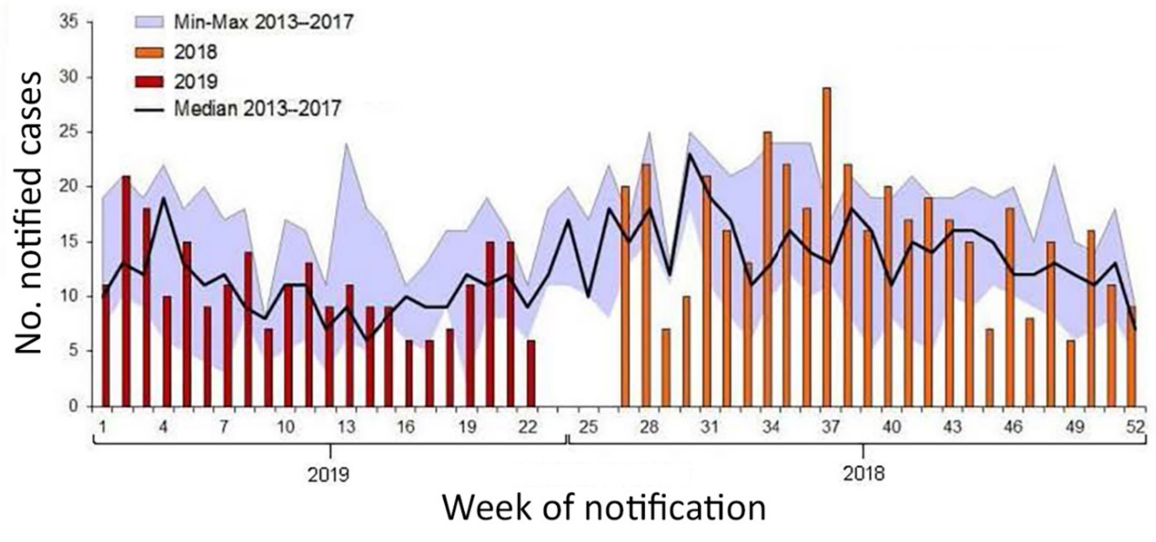

Appendix Figure 1. Number of notified listeriosis cases during the 2018–2019 outbreak in Germany compared with minimum, median, and maximum reported case numbers per week during the reference period, 2013–2017.

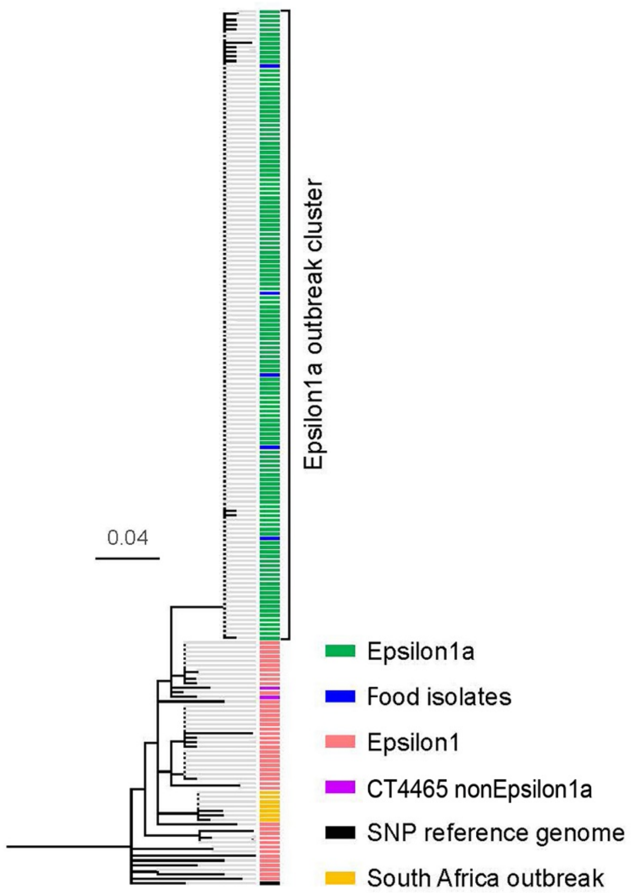

Appendix Figure 2. Maximum likelihood tree of *Listeria monocytogenes* Epsilon1a outbreak cluster, Germany. SNP calling demonstrated phylogenetic relatedness of isolates after read mapping to the genome of the *L. monocytogenes* serogroup IVb reference strain 10-092876-0769 LM12 (*2*). SNP, single nucleotide polymorphism. Scale bar indicates nucleotide substitutions per site.

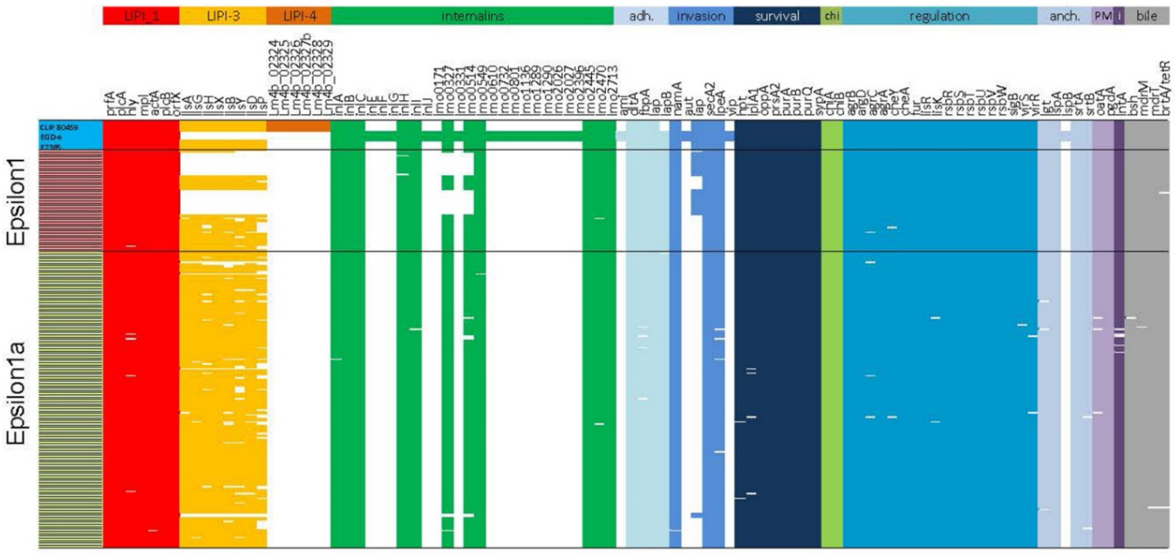

Appendix Figure 3. Virulome analysis of *Listeria monocytogenes* Epsilon1 and Epsilon1a isolates. Assembled genome sequences were searched for genes belonging to LIPI-1, LIPI-3, or LIPI-4; encoding internalins; or involved in adhesion, invasion, intracellular survival, chitin hydrolysis, gene regulation, protein anchoring to the cell surface, peptidoglycan modification, immunomodulation, or bile resistance. Genomes of strains EGD-e (accession no. NC_003210.1), F2365 (accession no. NC_002973.6), and CLIP80459 (accession no. NC_012488.1) were included for comparison. The variability in the presence or absence of chromosomally encoded virulence genes within the highly clonal Epsilon1a cluster likely is not real, but instead results from acceptance bias due to cutoff values used during allele calling. adh, adhesion; anch, protein anchoring; chi, chitin hydrolysis; I, immunomodulation; LIPI, *Listeria* pathogenicity island; PM, peptidoglycan modification.

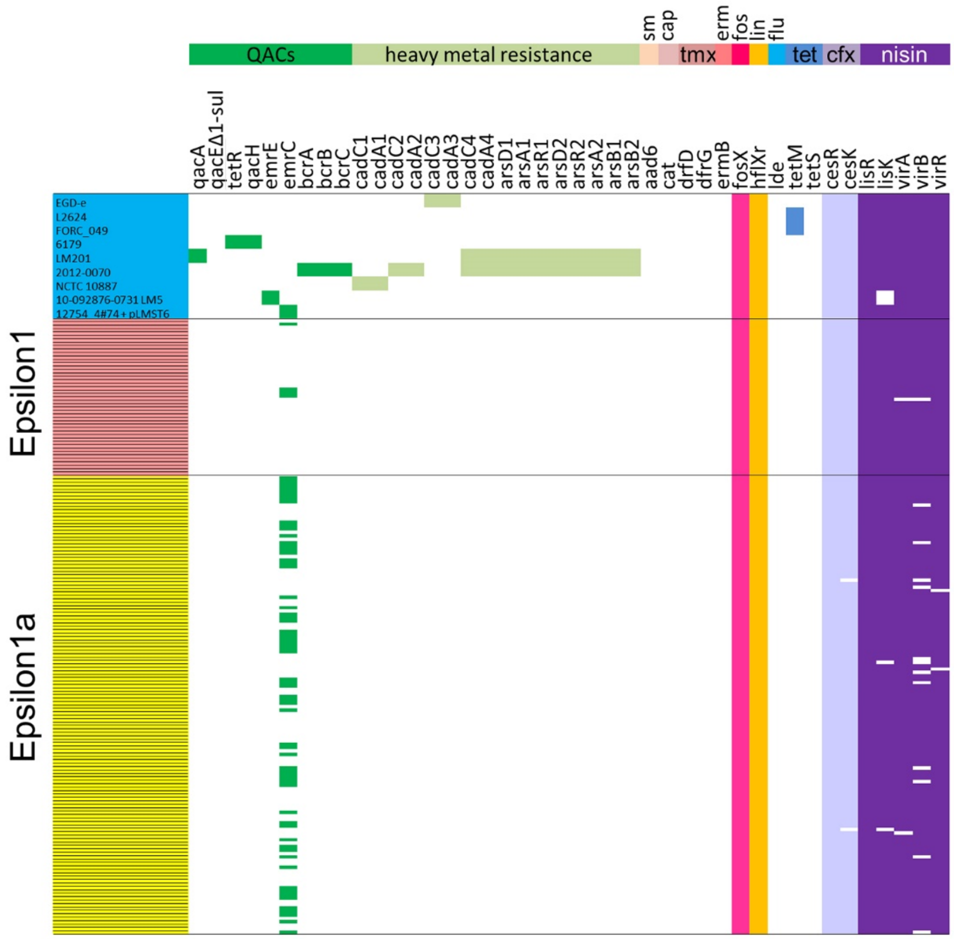

Appendix Figure 4. Resistome analysis of clinical *L. monocytogenes* Epsilon1 and Epsilon1a isolates. Genes are listed at the top. Assembled genome sequences were searched for genes known to confer resistance to sanitizers, heavy metals, and antimicrobial drugs by using a task template in SeqSphere (Ridom, https://www.ridom.de). Genomes of strains EGD-e (accession no. NC_003210.1), L2624 (accession no. NZ_CP007686), FORC_049 (accession no. NZ_CP016629), 6179 (accession no. NZ_HG813249), LM201 (accession no. SAMN03267130), 2012–0070 (accession no. SAMN05715911), NCTC 10887 (accession no. SAMN06434199), 10–092876–0731 LM5 (NZ_CP019618), and 12754_4#74 (accession no. ERR564017), and pLMST6 (accession no. GCA_900164035.1) were included for comparison. Of note, the *emrC* gene is located on plasmid pLMST6 that occurs in certain ST6 strains (*2*). Plasmid loss might explain why it was not detected throughout the entire Epsilon1a population. cap, chloramphenicol; cfx, ceftriaxone; erm, erythromycin; flu, fluoroquinolones; fos, fosfomycin; lin, lincomycin; QACs, quaternary ammonium compounds; sm, streptomycin; tet, tetracycline, tmx, trimethoprim.
